# Phosphorylation of FOXP3 by LCK Downregulates MMP9 Expression and Represses Cell Invasion

**DOI:** 10.1371/journal.pone.0077099

**Published:** 2013-10-14

**Authors:** Kumiko Nakahira, Akihiro Morita, Nam-Soon Kim, Itaru Yanagihara

**Affiliations:** 1 Department of Developmental Medicine, Research Institute, Osaka Medical Center for Maternal and Child Health, Izumi, Osaka, Japan; 2 Biomedical Genomics Research Center, Korea Research Institute of Bioscience and Biotechnology, Yuseong-gu, Daejeon, Korea; Hungarian Academy of Sciences, Hungary

## Abstract

Forkhead Box P3 (FOXP3) is a member of the forkhead/winged helix family of the transcription factors and plays an important role not only as a master gene in T-regulatory cells, but also as a tumor suppressor. In this study, we identified lymphocyte-specific protein tyrosine kinase (LCK), which correlates with cancer malignancy, as a binding partner of FOXP3. FOXP3 downregulated LCK-induced *MMP9*, *SKP2*, and *VEGF-A* expression. We observed that LCK phosphorylated Tyr-342 of FOXP3 by immunoprecipitation and *in vitro* kinase assay, and the replacement of Tyr-342 with phenylalanine (Y342F) abolished the ability to suppress MMP9 expression. Although FOXP3 decreased the invasive ability induced by LCK in MCF-7 cells, Y342F mutation in FOXP3 diminished this suppressive effect. Thus we demonstrate for the first time that LCK upregulates FOXP3 by tyrosine phosphorylation, resulting in decreased MMP9, SKP2, and VEGF-A expression, and suppressed cellular invasion. We consider that further clarification of transcriptional mechanism of FOXP3 may facilitate the development of novel therapeutic approaches to suppress cancer malignancy.

## Introduction

Forkhead box transcriptional factor families are involved in the network of post-translational modifications, including phosphorylation and protein–protein interactions, which provide an integrated cellular response to changes in the physiological status [1–3]. Forkhead box P3 (FOXP3) is a forkhead/winged-helix family member. *FOXP3* was originally identified as the causative gene for immune dysregulation, polyendocrinopathy, and enteropathy with X-linked (IPEX) inheritance [4,5]; in addition, it is the master gene for T-regulatory cells [6]. FOXP3 interacts with other transcription factors, including a nuclear factor of activated T cells [7,8], a nuclear factor kappa-B (NF-κB) [8], and an acute myeloid leukemia 1 [9], and blocks their ability to induce endogenous target gene expression, such as *interleukin 2*, *interleukin 4*, and *interferon-gamma*. Recent reports reveal that FOXP3 expression appears widespread in normal epithelia and aberrant in various solid tumors including breast cancer [10], ovarian cancer [11], prostate cancer [12], and pancreatic carcinoma [13] and cells lines of colon cancer [14]. FOXP3 acts as a transcriptional repressor of oncogenes, *HER-2/ErbB2* [10] and S-phase kinase-associated protein 2 (SKP2) [15], and FOXP3-regulated microRNAs suppress special AT-rich sequence-binding protein 1 [16], whereas deletions of FOXP3 exons extinguish those suppressive function in a breast cancer cell line [10]. Although tumor suppression by FOXP3 has been investigated by many researchers, regulatory proteins that functionally modify FOXP3 are still unknown. 

Lymphocyte-specific protein tyrosine kinase (LCK), a member of the Src family of non-receptor protein tyrosine kinases, is mostly expressed in T cells, normal breast tissue, and breast cancer tissue and cell lines [17]. LCK is activated under hypoxia/reoxygenation conditions by phosphorylation of Tyr-394 [18–20]. In the human breast cancer cell line, MCF-7, and in breast cancer samples, cross-talk between LCK and the protein tyrosine kinase syk plays a role in upregulating urokinase-type plasminogen activator (uPA) and matrix metalloproteinase 9 (MMP9) expression, which are associated with invasion and metastasis [21], via Sp1 transcription factor (SP1) activation under the hypoxia/reoxygenation conditions [22]. LCK also induces the nuclear translocation of NF-κB in MCF-7 cells, which also activates uPA expression following hypoxia/reoxygenation [23]. These findings suggest the involvement of LCK as a key regulator in breast cancer malignancy and/or tumor metastasis. 

It has been unclear whether FOXP3 is regulated by post-translational modifications. Recently it has been reported that cyclin-dependent kinase 2 phosphorylates FOXP3, and negatively regulates stability and activity of FOXP3 [24]. 

In this report, we demonstrate that LCK phosphorylates FOXP3 in MCF-7 cells, and MMP9 expression is regulated by phosphorylation of Tyr-342 of FOXP3 by LCK. 

## Materials and Methods

### Constructs

A cDNA encoding full-length human FOXP3 (amino acids 1–431: Full) or a fragment with a truncated forkhead domain (amino acids 1–332: ∆FKH) was inserted into a maltose-binding protein (MBP) fusion vector pMAL-c2 (NEB) at the *Bam*HI and *Sal*I sites, and into the GAL4 DNA binding domain fusion vector pGBKT7 (Clontech) at the *Nde*I and *Bam*HI sites. A cDNA encoding FOXP3-Full or mutated FOXP3 (described below in “Mutagenesis”) was inserted into the mammalian expression vector p3×FLAG-CMV-14 (Sigma-Aldrich) at the *Eco*RI and *Bam*HI sites. A cDNA encoding full-length human LCK or mutated LCK was inserted into pET-32a (Novagen) at the *Bam*HI and *Xho*I sites, and the mammalian expression vector pcDNA4/myc-His (Invitrogen) at the *Eco*RI and *Xho*I sites. Plasmids were transfected into MCF-7 cells using Fugene® 6 Transfection Reagent (Roche). 

### Mutagenesis

FOXP3 Y191F, Y330F, Y342F, and Y364F and LCK Y505F mutations were introduced using a Stratagene QuikChange site-directed mutagenesis kit (Stratagene) with double stranded oligonucleotides. Forward and reverse oligonucleotide primers were 5′- ccagagctccttcccactgctgg-3′ and 5′-ccagcagtgggaaggagctctgg-3′ for FOXP3 Y191F; 5′-caacatggacttcttcaagttcc-3′ and 5′-ggaacttgaagaagtccatgttg-3′ for FOXP3 Y330F; 5′-ctttcaccttcgccacgctcatc-3′ and 5′-gatgagcgtggcgaaggtgaaag-3′ for FOXP3 Y342F; 5′-caatgagatcttccactggttcac-3′ and 5′-gtgaaccagtggaagatctcattg-3′ for FOXP3 Y364F; and 5′-GAGGGCCAGTTCCAGCCTCAG-3′ and 5′-CTGAGGCTGGAACTGGCCCTC-3′ for LCK Y505F. 

### Cell culture

Human breast cancer cell line MCF-7 was obtained from the human health science research resources bank (Japan), and was maintained in Dulbecco’s modified Eagle’s medium (DMEM) supplemented with 10% FCS, 100 units/mL penicillin, and 0.1 mg/mL streptomycin in a humidified atmosphere containing 5% CO_2_ and 95% air at 37 °C. For LCK inhibition assay, cells were pretreated with LCK inhibitor, PP2 (10 μM) (Sigma-Aldrich) or emodin (20 μM) (Sigma-Aldrich).

### Generation of an anti-pTyr-342–FOXP3 antibody

The phospho-Tyr-342-specific antiserum was raised against a chemically synthesized phosphopeptide C+(PEG Spacer)+RPPFTpYATLIR (Scrum Inc.). Antiserum from a rabbit immunized with the phosphopeptide was further affinity-purified using phosphopeptide-conjugated sepharose. Subsequently, to remove antibodies that recognize the unphosphorylated peptide, the affinity-purified anti-pTyr-342-FOXP3 antibody was passed through a column conjugated with unphosphorylated peptide C+(PEG Spacer)+RPPFTYATLIR. The purified antibody strongly reacted with the phosphopeptide, but not with the unphosphorylated peptide (data not shown). 

### Yeast two-hybrid assay

FOXP3 ∆FKH was used to screen a human thymus cDNA library in the yeast two-hybrid assay using the Matchmaker^TM^ two-hybrid system 3 (Clontech laboratories, Inc.) according to the manufacturer’s instructions. About 9.6 × 10^5^ transformants were screened, and library plasmids from 140 positive clones were analyzed using DNA sequencing. β-galactosidase activity was measured using liquid and filter assays.

### Immunoprecipitation and immunoblot analysis

Cell lysates were prepared in a lysis buffer (1% NP-40, 0.5 mM EDTA, 1:1000 diluted Protease Inhibitor Cocktail Set III (Calbiochem)/PBS or 20 mM Tris pH 7.5, 150 mM NaCl, 1% NP-40, 1 mM Na_3_VO_4_, 20 mM NaF, 1:1000 diluted Protease Inhibitor Cocktail Set III). Soluble proteins were subjected to immunoprecipitation with anti-Myc (MBL) or anti-FLAG (Sigma-Aldrich) antibodies. An aliquot of the total lysate was included as a control. Immunoblot analysis was performed using the anti-Myc, anti-FLAG, anti-pTyr, PY-20 (Sigma) and 4G10(Millipore), or anti-pTyr-342-FOXP3 antibodies. The antigen–antibody complexes were visualized using Western Lightning® Plus-ECL (Perkin-Elmer). 

### MBP pull-down assay

MBP alone and MBP fusion proteins were expressed and purified using Amylose Resin (NEB) according to the manufacturer’s instructions. His-LCK was expressed and purified using chelating sepharose fast flow (GE Healthcare) according to the manufacturer’s instructions. After binding of MBP, MBP-FOXP3 ∆FKH, or MBP-FOXP3-Full to the amylose resin, His-LCK was incubated with 50 μL of the beads in a buffer (20 mM HEPES pH 7.4, 0.5% NP-40, 100 mM NaCl) for 2 h at 4 °C. After washing, proteins were eluted with 10 mM maltose and analyzed by immunoblotting using an anti-His-Tag (27EB) antibody (cell signaling) or an anti-MBP antibody (NEB).

### Confocal microscopy

Cells plated on gelatin-coated coverslips were transfected with p3×FLAG-CMV-14-*FOXP3* and pcDNA4/myc-His-*LCK*. At 48 h post-transfection, cells were fixed in 4% paraformaldehyde for 15 min, and permeabilized with 0.1% Triton X-100/PBS for 10 min. After treatment for blocking by 10% FCS/PBS for 30 min, cells were incubated with goat anti-FOXP3 (abcam) and mouse anti-LCK (Sigma) antibodies in 10% FCS/PBS overnight at 4 °C. Subsequently, cells were incubated with Alexa Fluor 546 donkey anti-goat IgG (Invitrogen) and Alexa Fluor 488 goat anti-mouse IgG (Invitrogen) in10% FCS in PBS for 2 h at 4 °C. Laser scanning confocal imaging system (Fluoview FV500, OLYMPUS) was used to visualize the sub-cellular localization of FOXP3 and LCK.

### Gene expression analyses

Levels of *MMP9*, glyceraldehyde-3-phosphate dehydrogenase (GAPDH), *SKP2*, vascular endothelial growth factor A (VEGF-A), and *18S ribosomal RNA* (*18S rRNA*) expression were quantified using real-time polymerase chain reaction (PCR). Total RNA was isolated from the cells transfected with several plasmids using RNeasy® Mini Kit (Qiagen), which were treated with RNase-free DNase (Qiagen) according to the manufacturer’s instructions. Total RNA was then reverse-transcribed into cDNA using PrimeScript^TM^ 1^st^ strand cDNA Synthesis Kit (Takara) according to the manufacturer’s instructions. cDNAs were mixed with a QuantiTect^TM^ SYBR Green PCR Master Mix (Qiagen) and the following primers. Forward and reverse oligonucleotide primers were 5′-GGGCTTAGATCATTCCTCAGTGCC-3′ and 5′-GAAGATGTTCACGTTGCAGGCATC-3′ for MMP9 [25]; and 5′-CGGGAAGCTTGTCATCAATGG-3′ and 5′-GGCAGTGATGGCATGGACTG-3′ for GAPDH. Real-time PCR was performed using CHROMO 4^TM^ Continuous Fluorescence Detector (Bio-Rad). PCR conditions for MMP9 were 20 sec at 95 °C, 30 sec at 64 °C and 30 sec at 72 °C with 45 cycles, and for GAPDH were 20 sec at 95 °C, 30 sec at 55 °C and 30 sec at 72 °C with 35 cycles. PCR was performed with Taqman® Gene Expression Master Mix and Applied Biosystems primers (7500 Fast Real-Time PCR System: Applied Biosystems) for *SKP2* (catalog no. Hs01021864_m1), *VEGF-A* (Hs00900055_m1), and *18S rRNA* (Hs99999901_s1) according to the manufacturer’s instructions. 

### Gelatin zymography experiments

Zymography analysis was performed [26], media was replaced 48 h post-transfection and cells were cultured further for 48 h. The conditioned media were collected, and separated using 8% SDS-PAGE gel electrophoresis containing 0.1% gelatin. The gel was incubated in a zymogram renaturing buffer (2.5% Triton X-100) for 30 min at room temperature. The buffer was replaced with the zymogram developing buffer (50 mM Tris pH 7.6, 200 mM NaCl, 5 mM CaCl_2_, 0.02% Brij 35) and equilibrated for 30 min at room temperature. Subsequently, the media was replaced with fresh zymogram developing buffer, and incubated overnight at 37 °C. The gel was stained with 0.5% Coomassie brilliant blue R-250. Cell lysates were immunoblotted with an anti-actin antibody (Thermo Fisher Scientific). Each expression level was calculated using ImageJ (http://rsbweb.nih.gov/ij/), and MMP9 expression was normalized with actin levels. 

### *In vitro* kinase assay

Five μg of recombinant MBP, MBP-FOXP3-Full, or MBP-FOXP3 Y342A, and His-LCK proteins were incubated in 30 μl kinase assay buffer (20 mM HEPES pH 7.4, 100 mM NaCl, 0.1% NP-40, 10% glycerol, 5 mM MgCl_2_, 5 mM MnCl_2_, 150 mM NaF, 1:1000 diluted Protease Inhibitor Cocktail Set III, and 3 μM ATP), and then supplemented with 370 kBq γ-^32^P-ATP (GE Healthcare). After 20 min incubation at 30 °C, the resulting mixture was subjected to SDS-PAGE and ^32^P incorporation was measured using FLA-9000 (Fujifilm). 

### Cell invasion assay

The invasion assay was performed using 24-well BD Biocoat Matrigel invasion chamber with 8-μm polycarbonated filters (Becton Dickinson) [27]. Forty thousand cells, suspended in DMEM containing 5% FCS, were added to the upper chamber, and DMEM containing 20% FCS was placed in the lower compartment of the chamber. Cells were incubated for 26 h at 37 °C in a humidified incubator with 5% CO_2_. Non-migratory cells on the upper surface of the filter were removed by wiping with a cotton swab. Invasive cells that penetrated through the pores and migrated to the underside of the membrane were stained and counted under the light microscope. The invasion rate was calculated as the percentage of cells that invaded the matrigel compared with the cells that invaded the control insert. 

### Sequence and statistical analysis

Multiple sequence alignment of FOXP3 protein was performed with the ClustalW software (http://www.genome.jp/tools/clustalw/). Repeated measurements analysis of variance (ANOVA) with Tukey-Kramer post hoc comparisons were performed for multiple comparisons. 

## Results

### FOXP3 binds to LCK

**Figure 1:**
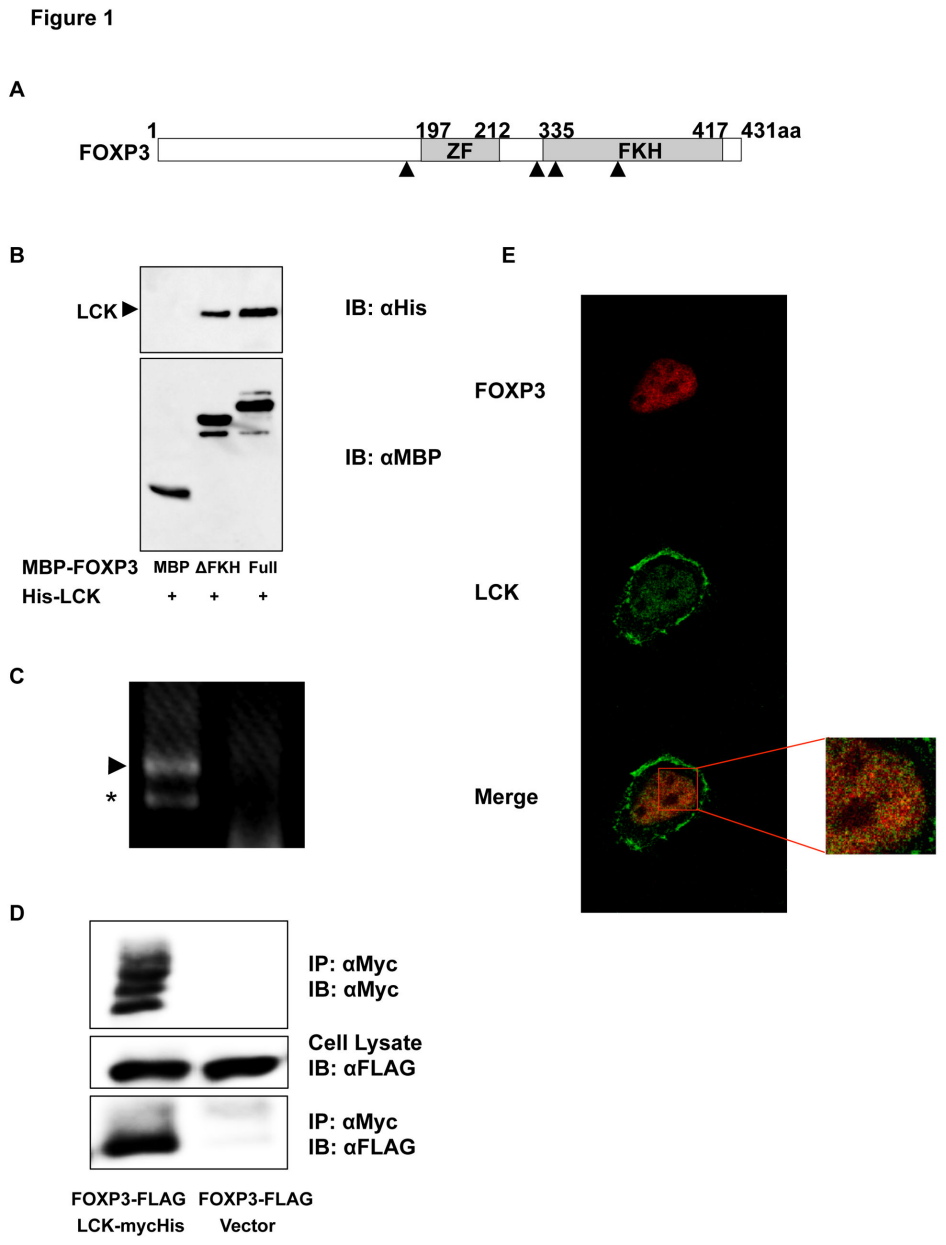
Identification of LCK as a binding protein of FOXP3. (A) Schematic representation of the domain architecture of FOXP3. ZF and FKH are C_2_H_2_ zinc finger and forkhead domains, respectively. Tyrosine residues of FOXP3 (Tyr-191, 330, 342, and 364) are represented by black triangles. (B) MBP pull-down assay. Recombinant His-tagged LCK bound to MBP-FOXP3 is depicted in the top panel. MBP and MBP-FOXP3 are depicted in the bottom panel. (C) Endogenous *LCK* expression in MCF-7 cells. The cDNA fragment of *LCK* from MCF-7 cells was amplified using PCR with primers covering from exon 6 to exon 10. Complete and exon 9-deleted amplicons in the left lane are represented by an arrowhead and an asterisk, respectively. The PCR product generated with total RNA from MCF-7 cells is shown in the right lane. (D) Co-immunoprecipitation of FOXP3 and LCK expressed in MCF-7 cells. Cell lysates were immunoprecipitated with an anti-Myc antibody and immunoblotted with anti-Myc (top) and anti-FLAG (bottom) antibodies. Cell lysates were immunoblotted with an anti-FLAG antibody (middle). Co-immunoprecipitated FOXP3 is depicted in the left lane of the bottom panel. (E) Co-localization of FOXP3 and LCK in MCF-7 cells. LCK partially co-localizes with FOXP3 in the nucleus (merge). Right panel shows the enlarged nucleus.

FOXP3 has C_2_H_2_ zinc finger and forkhead domains (Figure 1A). To identify FOXP3-interacting proteins, a yeast two-hybrid screening of a human thymus cDNA library was performed using a forkhead domain-truncated form of FOXP3, FOXP3 ∆FKH, as bait. One of the positive clones encoded the tyrosine kinase domain of human LCK (Table 1). To investigate whether FOXP3 could directly bind to LCK, a pull-down assay using recombinant MBP-FOXP3-Full or MBP-FOXP3 ΔFKH and His-LCK proteins was performed. As shown in Figure 1B, FOXP3 directly binds to LCK *in vitro*; the occurring interaction between FOXP3 ΔFKH and LCK reduced compared with FOXP3-Full and LCK. 

**Table 1:** Yeast two-hybrid assay.

		
DNA binding fusion	Transcriptional activation fusion	Units of β-galactosidase
FOXP3ΔFKH	clone (LCK)	3.08
FOXP3ΔFKH	Non-reactive clone	0.34
Positive control		10.0
Negative control		0.53

One of the positive clones resulting from screening with Foxp3 ∆FKH (1-332 amino acids) as a bait was identified as encoding a portion of LCK.

FOXP3 is reported to be a mammary tumor suppressor [10] and LCK is expressed in breast cancer tissues and cell lines [17]. We confirmed endogenous *FOXP3* expression and *LCK* expression (Figure 1C) using RT-PCR. MCF-7 cells expressed FOXP3 variants lacking exon 3, 3-4, 3 and 8, or 3-4 and 8 (data not shown). 

To confirm the interaction between FOXP3 and LCK in MCF-7 cells, co-immunoprecipitation assays and the immunocytochemical analysis of FOXP3 and LCK were performed. FOXP3 interacted with LCK in MCF-7 cells (Figure 1D); moreover, FOXP3 was predominantly localized to the nucleus, and LCK was localized both to the cell membrane and the nucleus (Figure 1E). In addition, LCK was found to be partially co-localized with FOXP3 in MCF-7 nucleus. 

### FOXP3 regulates human MMP9 expression

**Figure 2:**
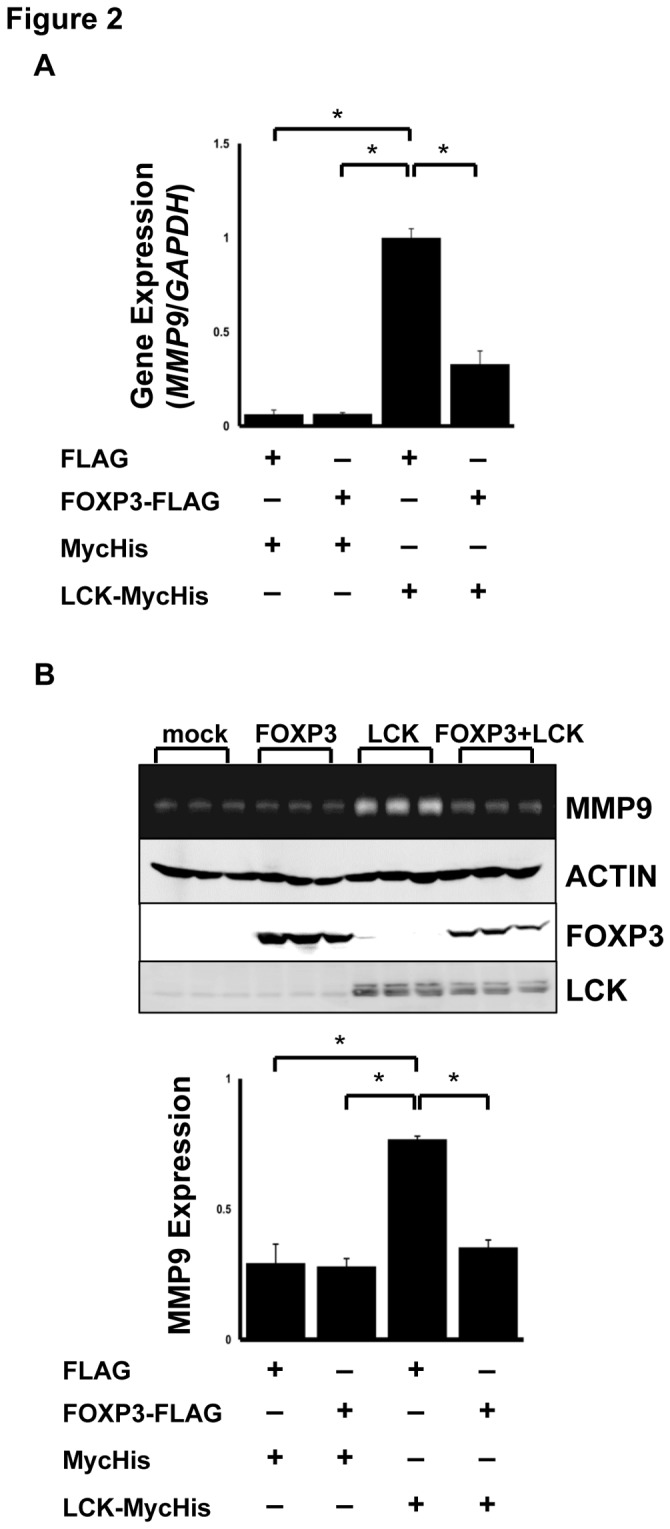
Regulation of human MMP9 expression by FOXP3. (A) Real-time PCR analysis of *MMP9* in MCF-7 cells. *MMP9* gene expression was determined using real-time PCR and normalized with *GAPDH* expression. (B) Zymography analysis. A gel image of MMP9 expression level (top). Each lane depicts independent samples from control, FOXP3, LCK, and FOXP3 and LCK-transfected cells, respectively. MMP9 expression was normalized with actin expression (bottom). The data represents the mean ± S.E. of three independent experiments. The asterisks indicate statistically significant differences (*p* < 0.05, Tukey-Kramer test).

As LCK upregulates MMP9 expression in MCF-7 cells [22], we examined the effect of FOXP3 on MMP9 expression in MCF-7 cells (Figure 2). Real-time RT-PCR analysis showed that mRNA levels of *MMP9* were 10-fold higher in LCK-transfected cells than in control cells 48 h post-transfection (Figure 2A). In contrast, the cells co-transfected with FOXP3 and LCK had significantly decreased *MMP9* expression compared with LCK-transfected cells. To examine the MMP 9 expression levels, conditioned media were subjected to a gelatin zymography assay (Figure 2B). In addition, LCK upregulated MMP9 expression at the protein level, while FOXP3 impaired LCK-induced MMP9 expression. 

### FOXP3 is phosphorylated by LCK

**Figure 3:**
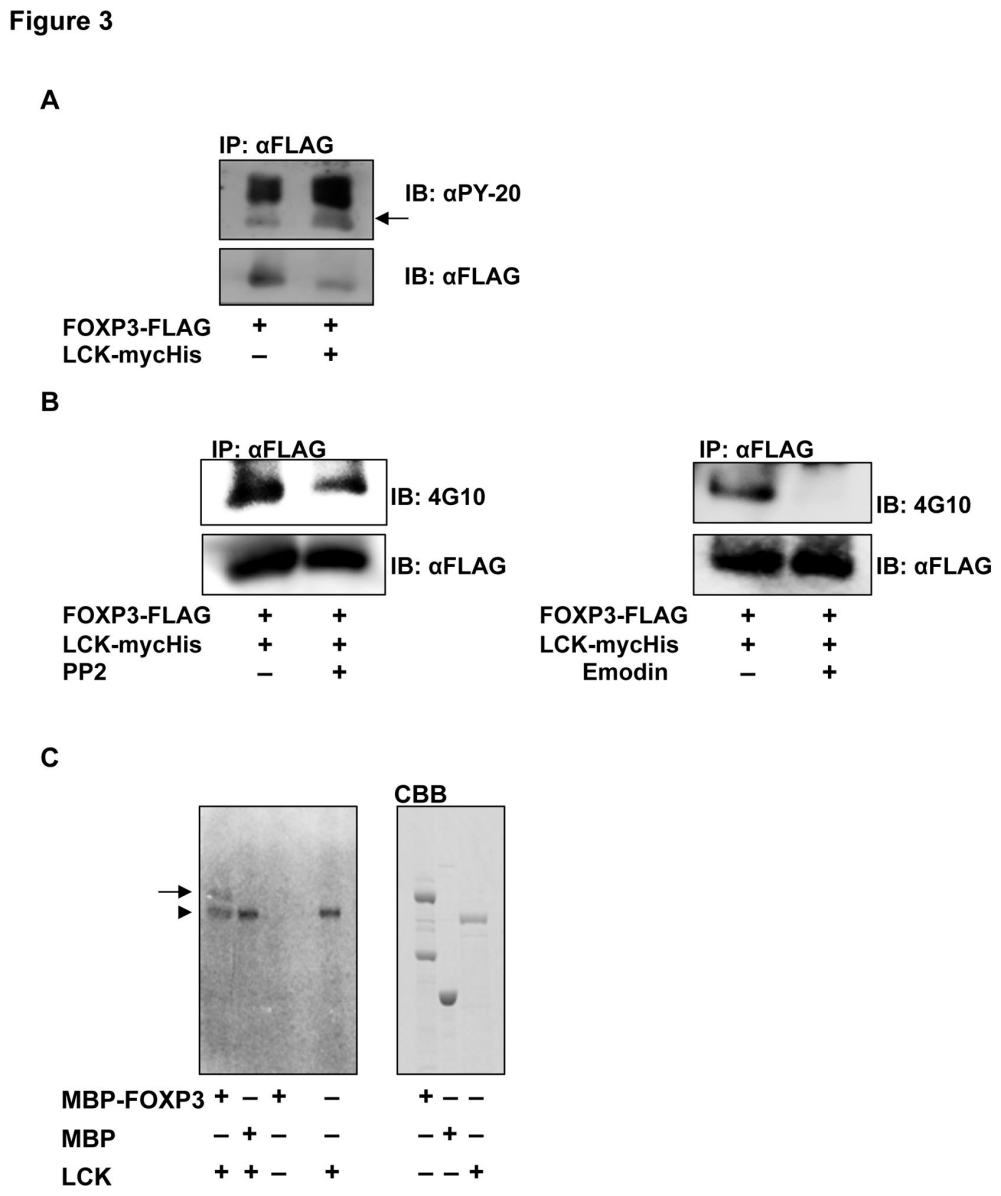
FOXP3 phosphorylation by LCK. (A) Phosphorylation of FOXP3 in MCF-7 cells. FOXP3 was immunoprecipitated with an anti-FLAG antibody and immunoblotted with an anti-PY-20 antibody (top). The antibodies were stripped and FOXP3-FLAG was detected using an anti-FLAG antibody (bottom). FOXP3 co-expressed with LCK was potently phosphorylated (arrow) compared with only FOXP3. (B) Decreased phosphorylation of FOXP3 by LCK inhibitors, PP2 (left) and emodin (right). Phosphorylated (top) and total (bottom) immunoprecipitated FOXP3 were detected using the indicated antibodies. Both PP2 and emodin inhibited the phosphorylation of FOXP3. (C) *In* *vitro* kinase assay. The recombinant proteins were incubated and separated using SDS-PAGE, and then autoradiographed (left). Right panel indicates each recombinant protein stained with CBB. Phosphorylated MBP-FOXP3 (arrow) was detected in the lane containing MBP-FOXP3 and LCK (an arrowhead).

As LCK is a tyrosine kinase, the phosphorylation of FOXP3 was investigated by Western blotting using the anti-pTyr antibodies, PY-20 and 4G10. We found that tyrosine phosphorylation level of FOXP3 increased in cells that co-expressed FOXP3 and LCK compared with cells that expressed only FOXP3 (Figure 3A). Treatment with PP2 or emodin, potent LCK inhibitors, blocked the LCK-mediated phosphorylation of FOXP3 (Figure 3B). These results indicate that FOXP3 is phosphorylated specifically in a LCK-dependent manner.

Subsequently, to examine whether FOXP3 is directly phosphorylated by LCK, an *in vitro* kinase assay was performed with recombinant MBP-FOXP3 and His-LCK proteins (Figure 3C). In addition to autophosphorylation of LCK, phosphorylation of FOXP3 was observed in the mixture containing His-LCK with MBP-FOXP3. These results revealed that FOXP3 is directly phosphorylated by LCK *in vitro*. 

### Tyr-342 phosphorylation of FOXP3 suppresses invasion of MCF-7 cells

**Figure 4:**
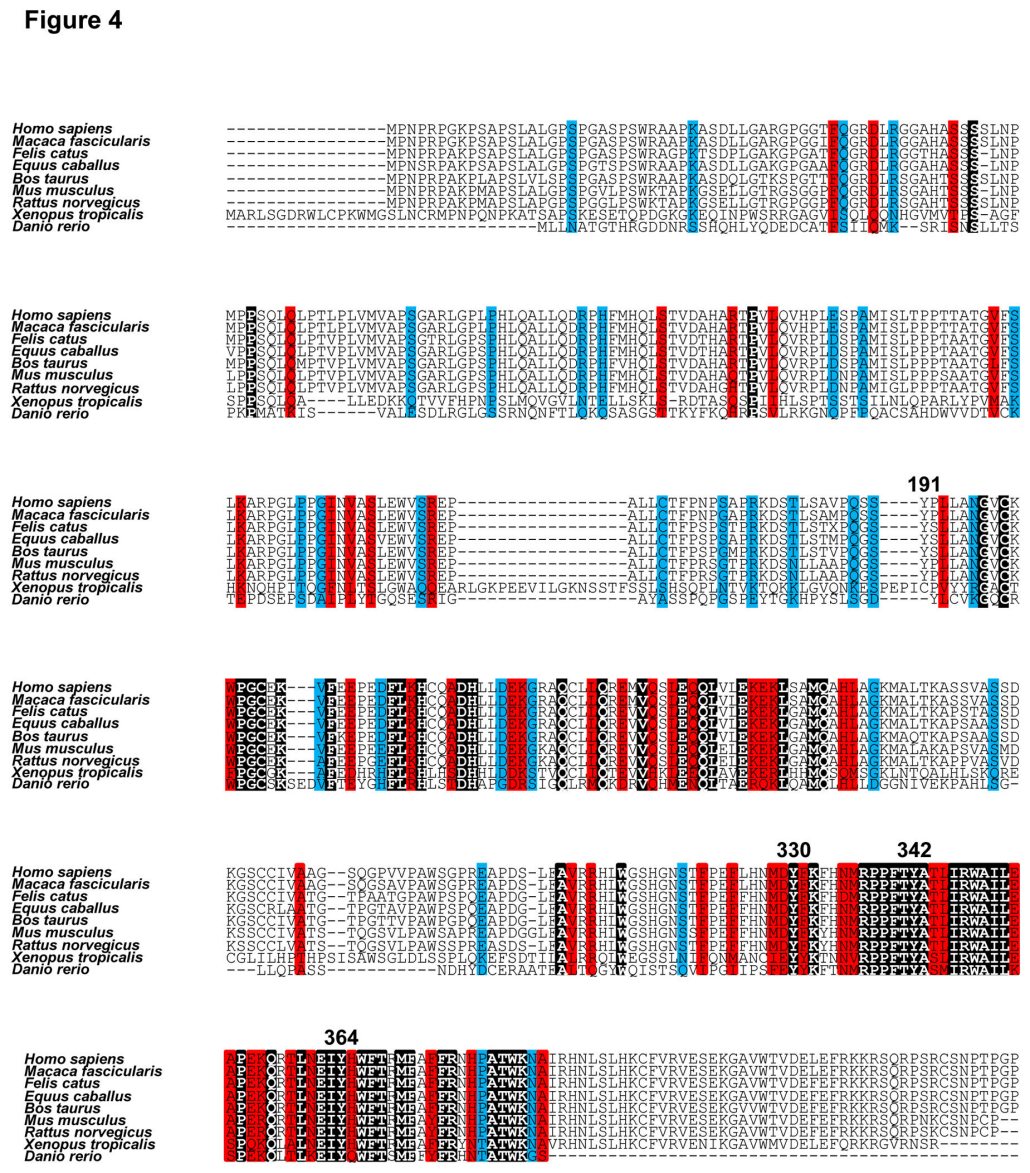
Alignment of amino acid sequence of FOXP3. ClustalW was used for the multiple alignment of amino acid sequence of FOXP3 from *Homo sapiens*, *Macaca fascicularis*, *Felis catus*, *Equus caballus*, *Bos taurus*, *Mus musculus*, *Rattus norvegicus*, *Xenopus tropicalis*, and *Danio rerio*. Black, red, and blue areas indicate identical, high, and low homologous amino acid residues, respectively. The tyrosine residues are represented by residue numbers.

**Figure 5:**
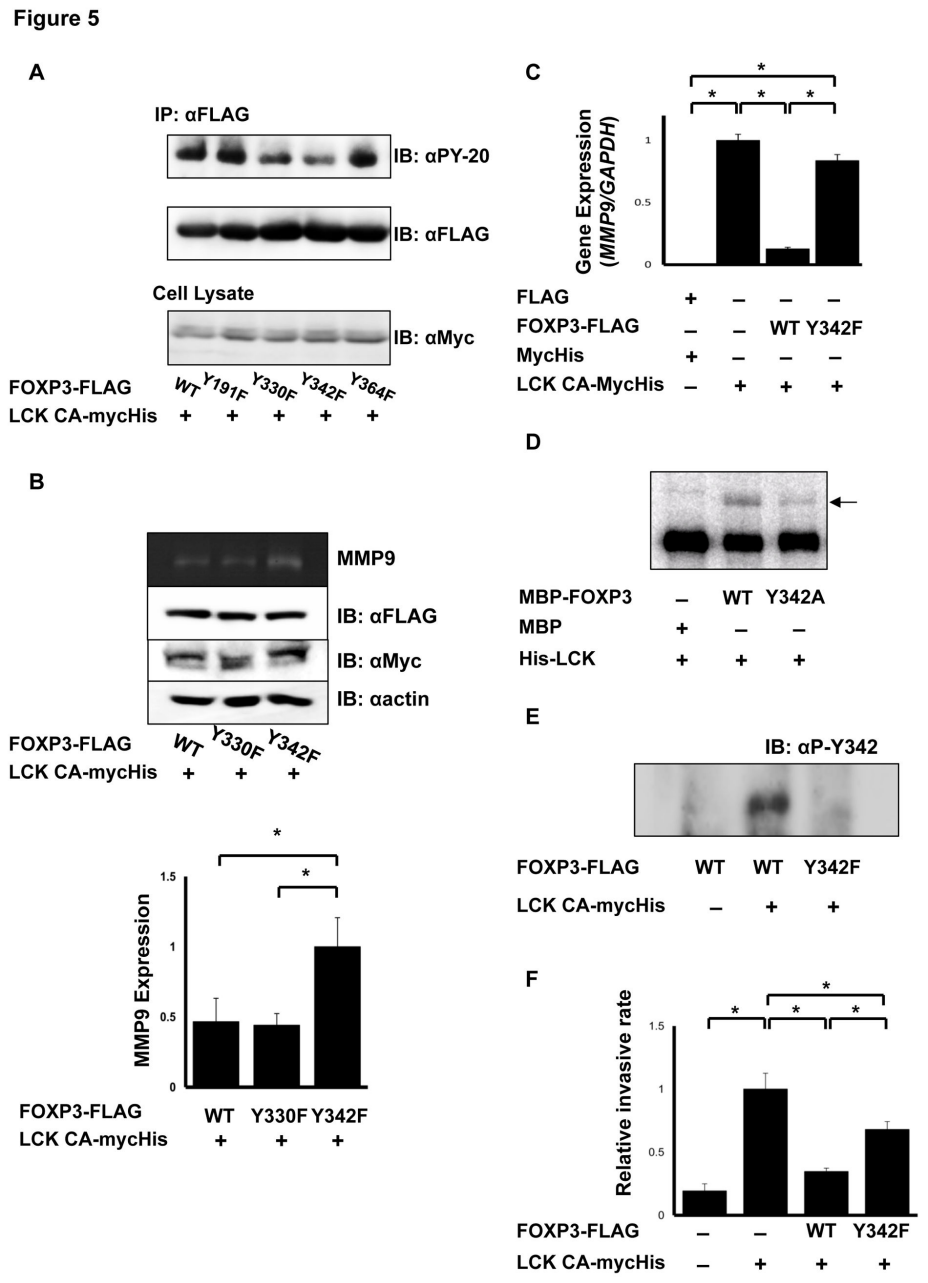
Correlation of phosphorylation at Tyr-342 of FOXP3 with transcriptional regulation. (A) Comparison of the phosphorylation levels of FOXP3 mutants. The level of phosphorylated (top) and total (middle) FOXP3 immunoprecipitated and constitutively-active mutant of LCK (LCK CA) in cell lysates (bottom) was detected using Western blotting. Phosphorylation of Y330F and Y342F mutants of FOXP3 was greatly decreased compared with FOXP3 WT. (B) Comparison of MMP9 expression regulated by FOXP3 mutant. MMP9 expression was analyzed using a zymography assay (top). FOXP3, LCK CA, and actin expression was determined using Western blotting (middle). MMP9 expression level was normalized with actin (bottom). FOXP3 Y342F mutant was unable to suppress MMP9 unlike FOXP3 WT and Y330F mutant. The data represents the mean ± S.E. of three independent experiments. The asterisks indicate statistically significant differences (*p* < 0.01, Fisher's LSD test). (C) Real-time PCR analysis of *MMP9* in FOXP3 Y342F expressing cells. *MMP9* expression in FOXP3 Y342F cells was significantly increased compared with FOXP3 WT cells. The data represents the mean ± S.E. of three independent experiments. The asterisks indicate statistically significant differences (*p* < 0.01, Tukey-Kramer test). (D) *In* *vitro* kinase assay. Analysis of phosphorylation of MBP-FOXP3 and MBP-FOXP3 Y342A (arrow) by LCK. Phosphorylation of MBP-FOXP3 (Y342A) was remarkably decreased compared with that of MBP-FOXP3 (WT). (E) Western blotting analysis using an anti-pTyr-342-specific antibody. FOXP3 and FOXP3 Y342F were immunoblotted with the anti-pTyr342 antibody. The antibody detected phosphorylation of FOXP3 only when LCK was co-transfected. (F) Levels of invasive cells. The cell number that invaded matrigel was normalized with cell counts that invaded the control insert. FOXP3 Y342F cells showed higher invasive rates than FOXP3 WT cells. The data represents the mean ± S.E. of six independent experiments. The asterisks indicate statistically significant difference (*p* < 0.01, Tukey-Kramer test).

Figure 4 depicts multiple sequence alignment of FOXP3 amino acid sequences from several species. FOXP3 possesses four tyrosine residues, Tyr-191, 330, 342, and 364. Biological significance of each tyrosine residue of FOXP3 was investigated using FOXP3 mutants, in which each tyrosine of FOXP3 was substituted with phenylalanine, with LCK constitutive active mutant (Y505F) [28]. Western blotting using a PY-20 antibody revealed that phosphorylation of FOXP3 Y330F and Y342F mutants was significantly decreased compared with wild-type FOXP3, suggesting that Tyr-330 and Tyr-342 of FOXP3 are phosphorylation targets of LCK (Figure 5A). Next, MMP9 expression in these mutants was analyzed. FOXP3 Y330F mutant suppressed MMP9 expression as well as FOXP3 WT (Figure 5B); however, the FOXP3 Y342F mutant lost its capability to suppress MMP9 expression (Figure 5B, C). These results demonstrated that FOXP3 suppresses MMP9 expression through the phosphorylation of Tyr-342 by LCK. Therefore, we focused on Tyr-342 residue in further experiments. An *in vitro* kinase assay using recombinant MBP-FOXP3 Y342A and His-LCK proteins showed that phosphorylation of MBP-FOXP3 Y342A protein significantly decreased compared with that of MBP-FOXP3 WT (Figure 5D). Moreover, whether Tyr-342 of FOXP3 was the specific phosphorylation site of LCK was investigated by generating a phospho-Tyr-342-specific (anti-pTyr-342-FOXP3) antibody. Western blotting using the anti-pTyr-342-FOXP3 antibody revealed that phosphorylation at Tyr-342 increased in cells that co-expressed FOXP3 and LCK, but not in cells that only expressed FOXP3 (Figure 5E), indicating that LCK specifically phosphorylates Tyr-342 of FOXP3. 

Because LCK enhances *uPA* and *MMP9*　expression resulting in cancer invasion and metastasis [22,23], the effect of FOXP3 WT and Y342F mutation on LCK-induced invasion was investigated. LCK Y505F-expressing cells had a 5-fold higher invasive ability than control cells, and co-expression of FOXP3 WT and LCK Y505F suppressed LCK Y505F-induced invasive activity (Figure 5F). Replacement of FOXP3 WT by FOXP3 Y342F partially restored the invasive behavior. Therefore, FOXP3 suppresses LCK-induced MMP-9 expression and the invasive ability through phosphorylation at Tyr-342 of FOXP3. 

### Tyr-342 phosphorylation of FOXP3 downregulates expression of LCK-induced genes

**Figure 6:**
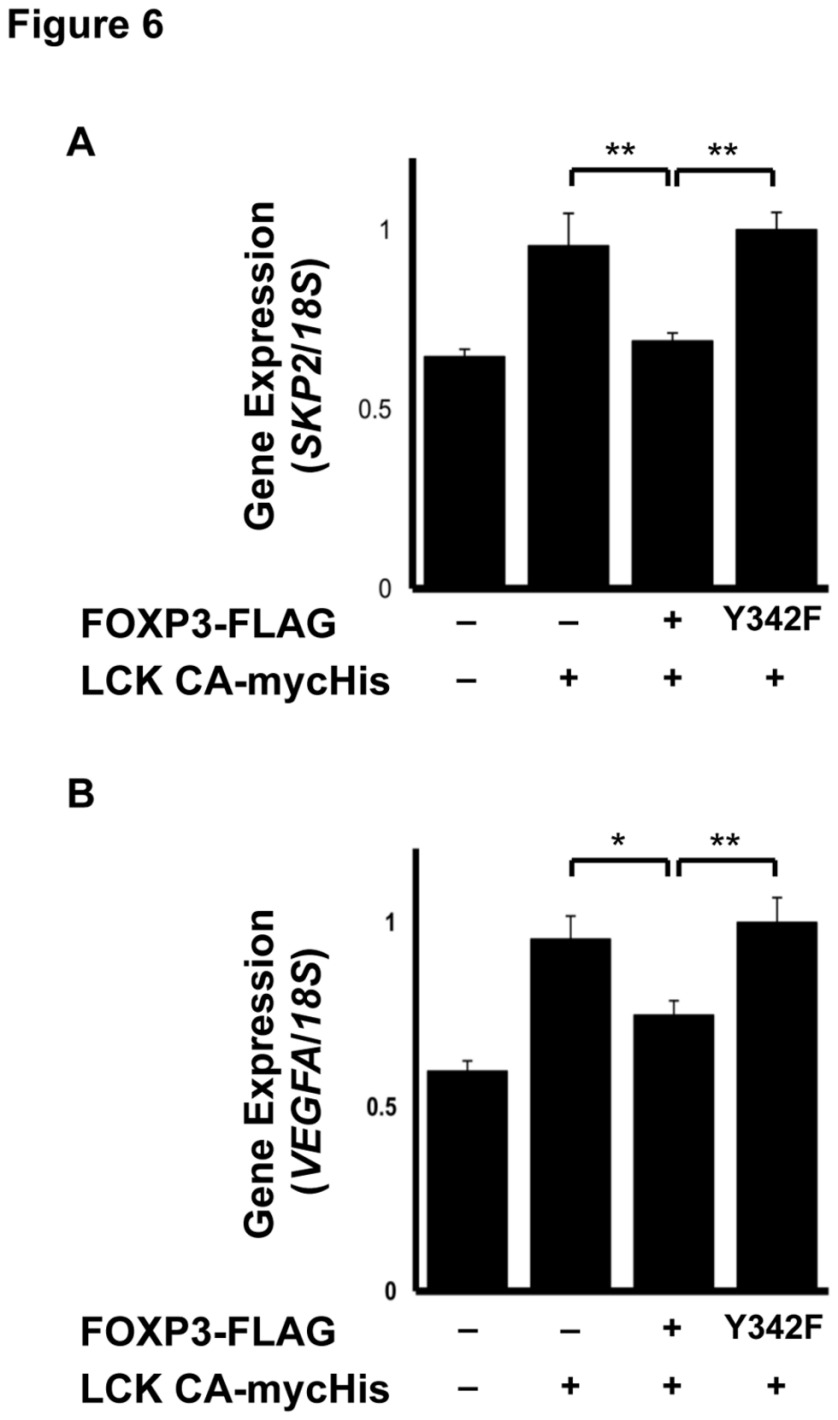
Real-time PCR analyses for *SKP2* and *VEGF-A*. (A) Real-time PCR analyses of *SKP2* expression and (B) *VEGF-A* expression. Gene expression was normalized with *18S* *rRNA* gene expression. FOXP3 WT suppressed the genes upregulated by LCK, while FOXP3 Y342F lost that capability. The data represents the mean ± S.E. of six independent experiments (*p < 0.05, *p < 0.01; Tukey-Kramer test). The data represents the mean ± S.E. of three independent experiments. The asterisks indicate statistically significant difference (*p* < 0.01, Tukey-Kramer test).

To examine whether FOXP3 suppresses other genes through phosphorylation, real-time PCR was performed for *SKP2* and *VEGF-A* levels involved in cancer malignancy. Expression of these genes slightly increased in LCK Y505F-transfected MCF-7 (LCK Y505F) cells, and their upregulation was suppressed in FOXP3 WT and LCK Y505F cells, but not in FOXP3 Y342F and LCK Y505F cells (Figure 6A, B). In summary, our data indicated that Tyr-342F phosphorylation of FOXP3 is involved in the inhibitory regulation of cancer malignancy by *SKP2*, *VEGF-A*, and *MMP9* expression. 

## Discussion

In this report LCK was identified as a binding protein of FOXP3 using a yeast two-hybrid assay, a pull-down assay, immunoprecipitation, and confocal microscopy in MCF-7 cells. Zuo et al. have demonstrated that MCF-7 cells express exon 3-4 lacking isoform of *FOXP3* [10]. We also confirmed that MCF-7 cells expressed four *FOXP3* isoforms lacking exon 3, 3-4, 3 and 8, and 3-4 and 8 (data not shown), supporting the idea that FOXP3 acts as a tumor suppressor. LCK is expressed not only in T cells but also in normal breast tissues and breast tumor samples. Endogenous *LCK* expression was observed using RT-PCR (Figure 1C), and LCK localized at the cell membrane and in the nucleus in MCF-7 cells (Figure 1E). Chakraborty et al. demonstrated LCK expression in the nucleus of MCF-7 cells as well as in normal and tumor breast tissues [22]. It was observed that LCK co-localized with FOXP3 in the nucleus (Figure 1E), suggesting that LCK could affect the transcriptional function of FOXP3. First, it was revealed that FOXP3 suppressed LCK-induced MMP9 expression in MCF-7 cells. Furthermore, it was found that FOXP3 decreased the invasive ability of MCF-7 cells, thereby potentially decreasing cancer malignancy. Our results clearly explain the function of FOXP3 as a tumor suppressor [10,11,15,16]. In fact, widespread deletions and somatic mutations of *FOXP3* were observed in breast cancer tissue [10]. As described above, FOXP3 lacks some exons in MCF-7 cells; thereby these variants could lose the ability to suppress gene expression involved in cancer malignancy.

To unravel the functional significance of the protein–protein interaction between FOXP3 and LCK, immunoprecipitation, *in vitro* kinase assays, and mutational analyses were performed. We observed that LCK phosphorylates Tyr-330 and Tyr-342 of FOXP3. Moreover, we revealed that Tyr-342 phosphorylation plays an important role in downregulating the MMP9, SKP2, which is essential for progression into mitosis in cell cycles [29] and suppressed by FOXP3 [15], and VEGF-A expression, which regulates tumor angiogenesis, and is upregulated by LCK [22], and the suppression of the invasive ability is enhanced by LCK. Tyr-342 of FOXP3 is encoded by exon 10. Therefore, the deletion mutants of FOXP3 lacking exon 3, and exon 3 and 8 expressed in MCF-7 cells might undergo Tyr-342 phosphorylation by LCK. However, these variants could not adopt a functional folding and thereby lack the tumor repressor activity. In this study, we revealed the phosphorylation of FOXP3 by LCK and the functional significance of this post-translational modification. We observed that FOXP3 Y342F mutant bound to LCK as well as FOXP3 WT (Figure S1), however, abolished its transcriptional repression activity. To elucidate the molecular mechanisms of transcriptional repression activity of FOXP3 by Tyr-342 phosphorylation, the precise structural analysis at atom level will be needed. 

To our knowledge, this is the first report demonstrating post-translational modification of FOXP3 through tyrosine phosphorylation involved in the transcriptional repression activity. LCK induces MMP9 expression mediated by NF-κB and SP1 transcription factors [22]. Furthermore, we found that the suppression of *SKP2* and *VEGF-A* expression, which are NF-κB target genes, was also associated with phosphorylation of Tyr-342 of FOXP3. Our observations and previous reports imply that gene suppression by Tyr-342 phosphorylation of FOXP3 may affect the function of NF-κB. Therefore, it was assumed that FOXP3 Y342F mutant could not bind to NF-κB thereby resulting in sustained NF-κB activation; however, the FOXP3 Y342F mutant bound to NF-κB as well as FOXP3 WT (Figure S2). Molecular mechanisms underlying transcriptional repression by FOXP3 might be regulated through multiple protein–protein interactions that include other proteins and/or additional post-translational modifications. Although our findings begin to clarify the inhibitory mechanisms of LCK-induced tumor related genes in MCF-7, detailed mechanisms remain to be elucidated in future studies. Anticancer drugs targeting Src family kinases have been developed. The present study reveals phosphorylation of FOXP3 by LCK, a Src family kinase that modulates tumor progression. Further clarification of FOXP3-functions may facilitate the development of novel therapeutic approaches targeting LCK-FOXP3 pathway to suppress cancer malignancy for personalized and selective targeted medicine.

## Supporting Information

Figure S1 **Co-immunoprecipitation of FOXP3 Y342F and LCK Y505F.** Cell lysates were immunoprecipitated with an anti-Myc antibody, and cell lysates and immunoprecipitants were immunoblotted with anti-Myc and anti-FLAG antibodies. Co-immunoprecipitated FOXP3 Y342F was detectable.
(TIF)

Figure S2 **Co-immunoprecipitation of FOXP3 derivatives and NF-κB.** Cell lysates were immunoprecipitated with an anti-NF-κB antibody, and cell lysates and immunoprecipitants were immunoblotted with anti-NF-κB and anti-FLAG antibodies. Co-immunoprecipitated FOXP3 WT, Y330F, and Y342F were detectable.
(TIF)
