# Supplementary figures and images for: Phosphorylation of FOXP3 by LCK Downregulates MMP9 Expression and Represses Cell Invasion
Source: PLoS One. 2013 Oct 14;8(10):e77099. doi: 10.1371/journal.pone.0077099 (PMC3796550; doi:10.1371/journal.pone.0077099)

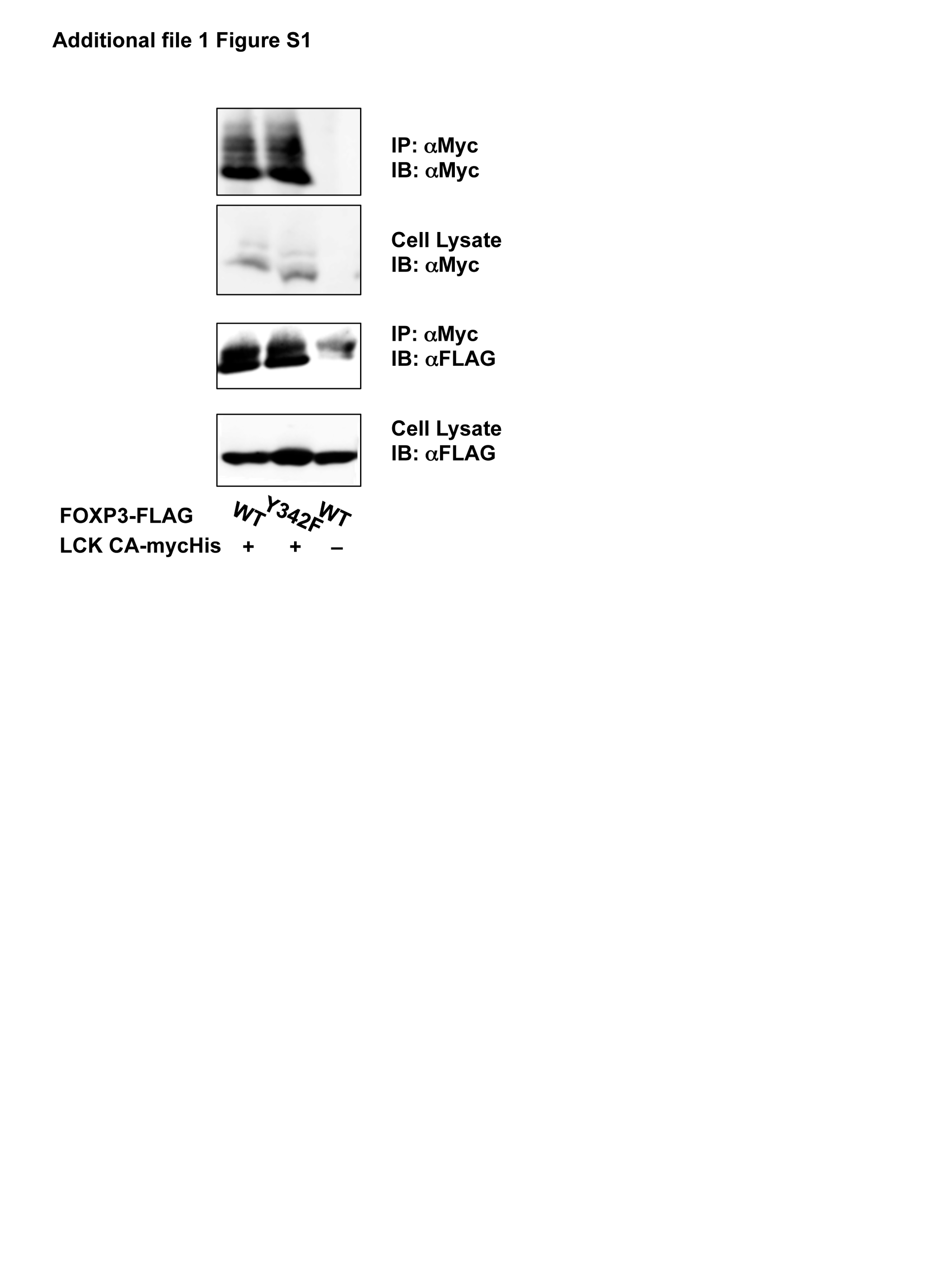

Supplement: Figure S1 — Co-immunoprecipitation of FOXP3 Y342F and LCK Y505F. Cell lysates were immunoprecipitated with an anti-Myc antibody, and cell lysates and immunoprecipitants were immunoblotted with anti-Myc and anti-FLAG antibodies. Co-immunoprecipitated FOXP3 Y342F was detectable. (TIF) [file pone.0077099.s001.tif]

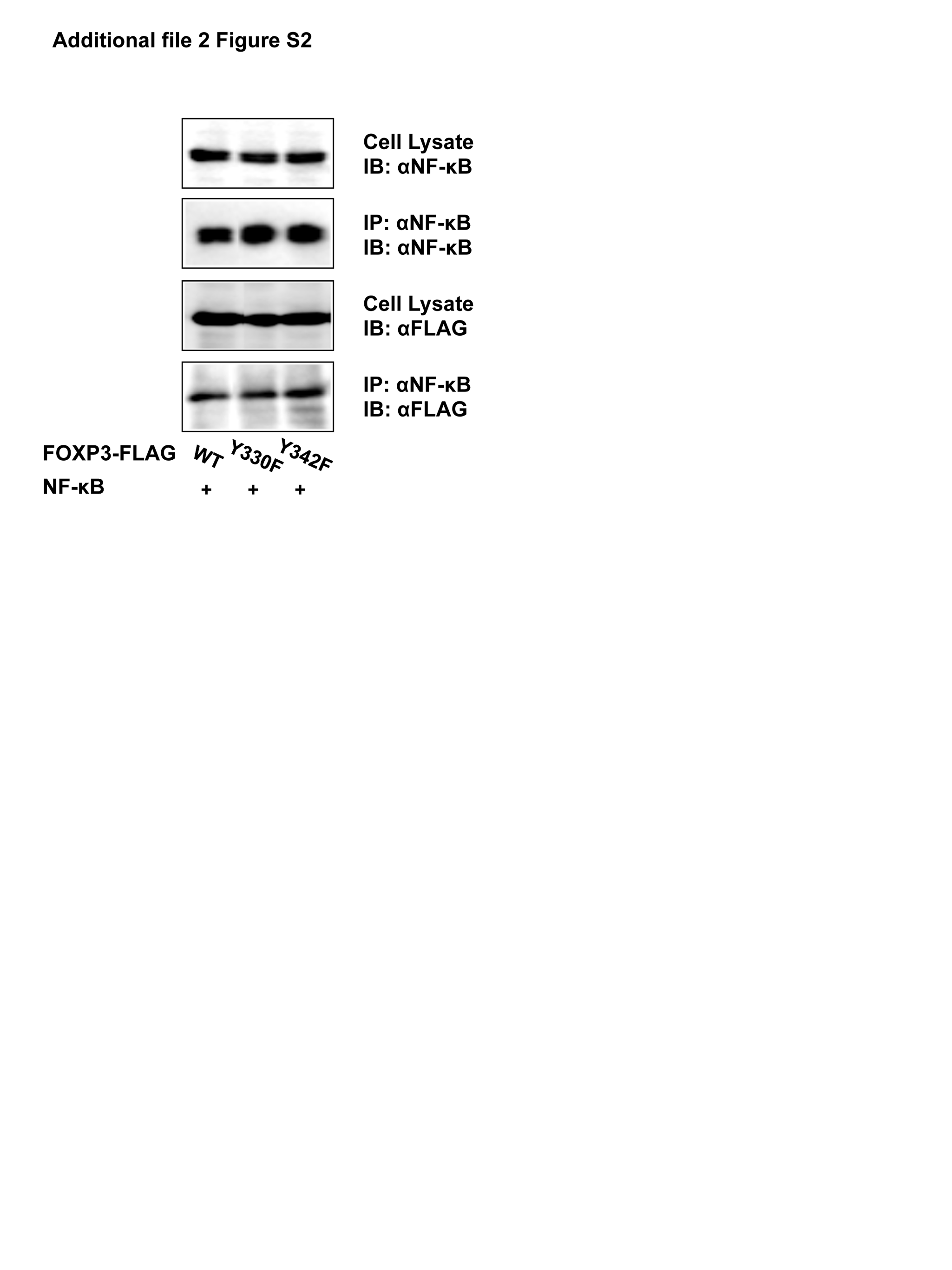

Supplement: Figure S2 — Co-immunoprecipitation of FOXP3 derivatives and NF-κB. Cell lysates were immunoprecipitated with an anti-NF-κB antibody, and cell lysates and immunoprecipitants were immunoblotted with anti-NF-κB and anti-FLAG antibodies. Co-immunoprecipitated FOXP3 WT, Y330F, and Y342F were detectable. (TIF) [file pone.0077099.s002.tif]
